# Phytochemical Profile, Antioxidant and Antidiabetic Activities of *Adansonia digitata* L. (Baobab) from Mali, as a Source of Health-Promoting Compounds

**DOI:** 10.3390/molecules23123104

**Published:** 2018-11-27

**Authors:** Alessandra Braca, Chiara Sinisgalli, Marinella De Leo, Beatrice Muscatello, Pier Luigi Cioni, Luigi Milella, Angela Ostuni, Sergio Giani, Rokia Sanogo

**Affiliations:** 1 Department of Pharmacy, University of Pisa, 56126 Pisa, Italy; alessandra.braca@unipi.it (A.B.); beatrice.muscatello@unipi.it (B.M.); pierluigi.cioni@libero.it (P.L.C.); 2 Research Centre for Nutraceutical and Healthy Foods “NUTRAFOOD”, University of Pisa, 56124 Pisa, Italy; 3 Department of Science, University of Basilicata, 85100 Potenza, Italy; chiara.sinisgalli@gmail.com (C.S.); luigi.milella@unibas.it (L.M.); angela.ostuni@unibas.it (A.O.); 4 Aide au Développement de la Médecine Traditionnelle (AIDEMET), ONG, Bamako, Mali; aidemet@afribonemali.net; 5 Département Médecine Traditionelle (DMT), INRSP, B.P. 1746 Bamako, Mali; rosanogo@yahoo.fr

**Keywords:** *Adansonia digitata*, baobab, phenols, tiliroside, volatile organic compounds, antioxidant activity, antidiabetic activity, LC-ESI-MS/MS

## Abstract

*Background*: *Adansonia digitata* L. (Malvaceae), also known as baobab, is a tree attracting recent interest especially due to the high nutritional value of the fruit pulp. However, few studies are reported on the secondary metabolite content, showing high variability depending on the geographic region. Methods: In this study, the chemical profiles of Malian commercial baobab fruits and leaves, focused on phenolic content, were investigated by HPLC coupled with a photodiode array (PDA)/UV and an electrospray ionization (ESI) mass spectrometer (MS) and gas chromatography (GC)/MS. In addition, the extracts of fruit pulps obtained from three different markets (Fruits 1, 2, and 3) were evaluated for their total phenolic content (TPC), antioxidant activity and α-glucosidase inhibition. Results: Baobab fruit pulps were found to be rich in procyanidins and flavonol glycosides, with tiliroside as the major constituent. The baobab leaves showed a similar profile respect to the fruits, but with more detected phenolics. All fruit pulp extracts exerted antioxidant activity (highest for Fruit 3) and higher α-glucosidase inhibition than acarbose used as standard. Conclusions: This study confirmed the variability of baobab with different origins and indicated Malian species baobab as a promising source of health-promoting substances.

## 1. Introduction

*Adansonia digitata* L. (Malvaceae), commonly called baobab, monkey bread or pharmacist tree, is a deciduous tree with wide distribution in most of Sub-Sahara semi-arid and sub-humid regions. In Mali, the areas of baobab populations are present in Sahelian and Sudanese agro-ecological zones, particularly in the regions of Ségou, Mopti, Sikasso, and Kayes. The species is usually associated with dry savannah, dry forests, and human settlements. In regions where baobab grows spontaneously, the tree constitutes an essential component of the agricultural landscape. It is a very long-lived tree with multi-purpose uses, providing food, shelter, clothing, ropes, manure and, last but not least, medicines, and cosmetics, as well as material for hunting and fishing [1,2,3].

The flour of baobab fruit pulp is popularly used to prepare refreshing drinks, sweets, but also sauces and, recently, ice creams. It is considered as a food complement, and can be recommended for the contribution to the daily intake of energy, carbohydrates and proteins for children and pregnant women [4,5].

The baobab fruit pulp is used in African traditional medicine as an antipyretic or febrifuge, anti-dysenteric, diaphoretic, immunostimulant, anti-inflammatory, analgesic, and probiotic remedy [1,2,3]. In Mali, baobab fruit pulp flour is also used to treat the diarrhoea of children and to stimulate the milk production in breastfeeding women. Baobab leaves are known in folk medicine as an antipyretic or febrifuge [2]. Biological studies reported that baobab fruit pulp exhibited antioxidant activity [6], hepatoprotective effect [7], cardioprotective [8], antidiabetic [9], and antitumor action [10].

In terms of macronutrients, baobab dried fruit pulp is low in fats and very high in fibers (about 50 g/100 g). The pulp has a low sugar content compared with other fruits. It is relatively low in proteins but is a good source of amino acids. Baobab dried fruit pulp would therefore be ideal as an ingredient for adding fibers to foods, as well as raising their overall nutritional profile. Its profile compared to other foods would indicate that it could be both a low glycaemic index food and a satiating ingredient, due to its high soluble fiber content [4,5].

Baobab fruit pulp contains more vitamin C than other fruits (over 100 mg/100 g) and it is a good source of calcium, iron, and magnesium [4,11]. Moreover, the baobab fruit pulp is a rich source of polyphenolic compounds that could play a protective role against oxidative stress [1,12]. Few studies are reported about chemical characterization of phenol compounds of baobab fruit pulp and results showed a high variability between plants from different geographic regions. A phytochemical study of a Nigerian baobab fruit pulp afforded to the isolation of hydroxycinnamic acid glycosides, iridoid glycosides, and phenylethanoid glycosides [13], that were also profiled by UHPLC-DAD-HR-ESI-MS technique. Very different results were reported by Sokeng et al. [14], that revealed the presence of procyanidins, phenolic acids, and flavonol glycosides in sample fruits from Cameroon by LC-ESI-MS/MS analysis. Tembo et al. [6] reported procyanidin B2, gallic acid, and epicatechin in a Malawi baobab fruit pulp. A great variation was observed also in nutritional value, possibly due to the origin, the quality, the manufacturing, the storage, etc. of studied samples, encouraging further investigations to improve scientific literature on this species [2]. Baobab leaves are also poorly investigated being phenolic acids, flavonoids, particularly quercetin and kaempferol glycosides and procyanidins, the main reported metabolites [9,14].

Taking into account the strong potential of baobab as functional food and the importance of this fruit in the African villages, as source of nutrients combating malnutrition and bioactive molecules, in this work we carried out qualitative and quantitative chemical analyses by HPLC coupled with a photodiode array (PDA)/UV detector and an electrospray ionization (ESI) mass spectrometer (MS), of fruit pulp originating from Mali, focusing the attention on the detection of secondary metabolites, in order to compare its chemical content with previous studies of fruits of different origin. Three sample fruits obtained from three Malian rural markets were selected and analyzed to investigate if commercial products shown differences in term of chemical composition, including both volatile and non-volatile compounds, probably due to processing methods. All sample fruits were also tested for their antioxidant and α-glucosidase inhibition activities, with the aim of improving the knowledge about beneficial effects of baobab on human health. In addition, since few studies are reported in the literature about constituents of baobab leaves, HPLC-PDA/UV-ESI-MS/MS analyses on Malian baobab leaves were also performed. 

## 2. Results and Discussion

### 2.1. Antioxidant Activity and α-Glucosidase Inhibition of Baobab Fruit Pulp

Baobab leaves and fruit are source of polyphenols important to reduce oxidative stress [14]. Previous studies reported mostly leaf antioxidant activity [9,15]. It has been shown a good scavenging activity vs. both DPPH (SC_50_ = 0.23 ± 0.01 mg/mL) and ABTS (IC_50_ = 1.87 ± 0.09 mmol/L TE/g) radicals. Moreover, they reported good capacity to reduce Fe^3+^ to Fe^2+^ (20.02 ± 1.83 mgGAE/g) [9] and also leaf extracts showed anti-inflammatory effect in LPS-stimulated RAW264.7, by reducing iNOS and NF-*κ*B expression, with possible involvement of other pathways [15,16]. Fruits are less investigated and in our study *n*-butanol extracts obtained from fruits of baobab purchased in three different local markets were tested to evaluate the content of polyphenols and in vitro antioxidant activity. Total phenolic content (TPC) values, measured as mg of Gallic Acid Equivalents (GAE)/g of extract, of *n*-butanol extracts ranged between 120.1 ± 4.7 and 161.40 ± 2.8 mgGAE/g of dried extract in Fruit 2 and Fruit 3, respectively (Table 1).

Antioxidant activity was evaluated through five different colorimetric methods. According to previous studies [6,17] baobab fruit extracts reported higher reducing power and radical scavenging activity toward synthetic radicals (DPPH and ABTS) (Table 1). Fruit 3 extract showed the highest activity in all the above mentioned tests. In fact, Fruit 3 reported 392.22 ± 28.13 mgTE/g and 799.44 ± 35.96 mgTE/g in DPPH and ABTS assays, respectively. Fruit 3 was the extract with the highest reducing power (458.50 ± 23.41 mgTE/g of dried extract) tested by FRAP assay (Table 1). Moreover, the antioxidant activity of fruit extracts was tested also towards superoxide anion, a physiological radical normally produced inside the body and on the inhibition of lipid peroxidation. The extract radical scavenging activity vs. superoxide anion was expressed as IC_50_, and Fruit 1 extract showed the highest antioxidant activity having the lowest value of IC_50_ (0.18 ± 0.02 mg/mL); Fruit 2 and Fruit 3 reported similar IC_50_ values (0.89 ± 0.09 and 0.55 ± 0.002 mg/mL). Interestingly, these values were lower than ascorbic acid used as standard (IC_50_ 0.95 ± 0.06 mg/mL), evidencing a promising health-promoting value of analyzed extracts. Comparing our results with those reported above, fruit extracts demonstrated to be effective as leaf extracts in the DPPH assay (our average SC_50_ is 0.15 mg/mL), or even more active, about one order of magnitude, in the ABTS or FRAP tests.

The oxidation of linoleic acid generates peroxyl free radicals, which will then oxidize the highly unsaturated β-carotene. The presence of antioxidants minimizes the oxidation of β-carotene. Fruit 3 reported the highest β-carotene bleaching inhibition activity (68.83 ± 0.38%) (Table 1).

**Figure 1:**
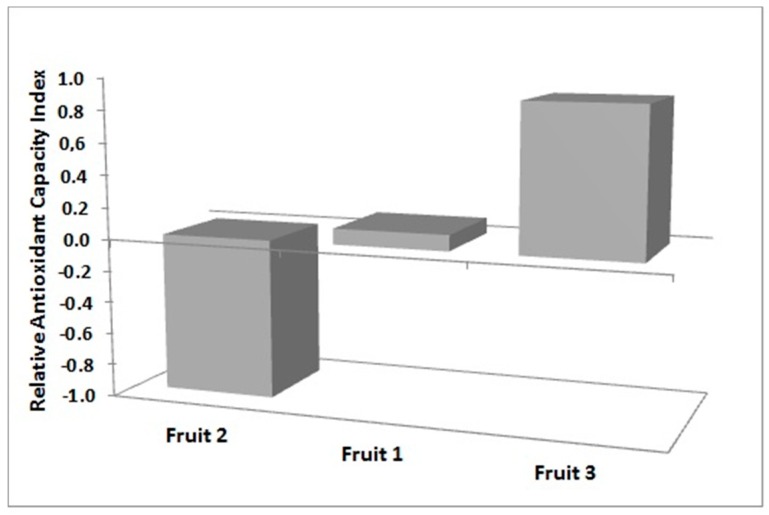
Relative antioxidant capacity index (RACI) values obtained comparing TPC, DPPH, FRAP, BCB, NO, and SO results. DPPH: 2,2-diphenyl-1-picrylhydrazyl; FRAP: ferric reducing antioxidant power; BCB: β-carotene bleaching assay; TPC: total polyphenolic content; RACI: relative antioxidant capacity index.

Relative antioxidant capacity index (RACI) was calculated among all the tested samples (Figure 1). RACI is an adimensional index that has been demonstrated to be a useful tool for comparison of results from different assays. In fact, RACI has been used to have a clear comparison among the antioxidant activity results obtained from DPPH, FRAP and BCB assays, and it has been calculated using Excel software (2010, Microsoft, Redmond, WA, USA). The values of antioxidant capacity in each data set are transformed into standard scores, derived by subtracting the mean from the raw data divided by the standard deviation [18]. The mean of standard scores was used for the RACI calculation. All antioxidant activity assays along with TPC were included in RACI calculation [19]. The TPC method was recently proposed for the determination of total reducing capacity of samples, which reflects the cumulative capacity of both phenolic and non-phenolic compounds to reduce the Folin-Ciocalteu reagent. According to previous results Fruit 3 had the highest RACI (0.90).

**Figure 2:**
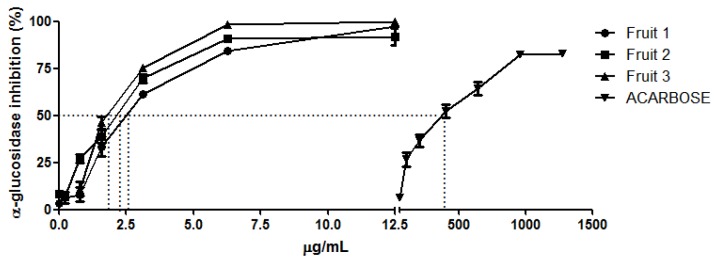
Concentration-dependent inhibition of α-glucosidase by baobab fruit pulp extracts and acarbose (standard).

**Figure 3:**
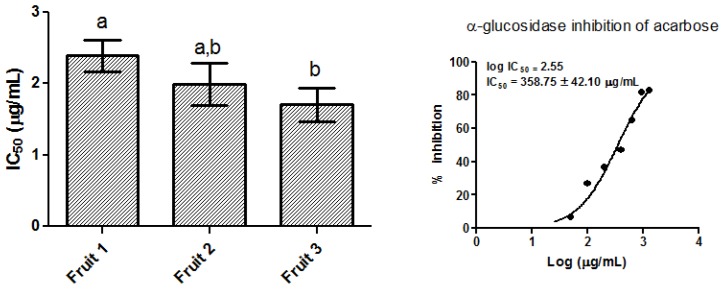
Inhibition of α-glucosidase by baobab fruit pulp extracts and acarbose (standard). Experiments were carried out in triplicate and data were reported as mean ± SD. Significant differences (*p* < 0.05) are highlighted with different letters (a, a,b and b).

The higher antioxidant activity could be due to polyphenols, such as proanthocyanidins and flavonol glycosides, identified in baobab fruit extracts [14,20,21]. Moreover, polyphenols can influence the activity of key enzymes. Many studies reported their ability to inhibit digestive enzymes such as α-glucosidase [19]. In vitro studies had shown that baobab fruit extracts interfere with starch degradation and reduce sugar release from starch-rich foods as bread [22]. However, there are no study that demonstrated inhibition of these enzymes by baobab fruit extracts, so together with the antioxidant activity measurement, we have also investigated *A. digitata* fruit extract on the in vitro α-glucosidase inhibitory activity. Glycosidases are widespread in microorganisms, plants, and animals. They are a very important class of enzymes, which catalyze a hydrolytic cleavage of glycosidic bonds in oligosaccharides or glycoconjugates. Among these glycosidases, α-glucosidase is able to catalyze the cleavage of glycosidic bonds involving terminal glucose connected at the site of cleavage through α-linkage at the anomeric center [23,24]. Glycosidases are involved in several important biological processes (like: digestion, biosynthesis of glycoproteins, and lysosomal catabolism of glucoconjugates) related to metabolic disorders and diseases, such as, diabetes, obesity, glycosphingolipid lysosomal storage disease, HIV infections, and tumors [25]. These observations indicate that the inhibition of glycosidases would represent a novel and important pharmacological approach towards the treatment of the above mentioned complications, including diabetes. Acarbose and fruit extracts were able to inhibit α-glucosidase in a dose dependent manner (Figure 2). Results, expressed as IC_50_, ranged from 1.71 ± 0.23 to 2.39 ± 0.22 μg/mL. All extracts reported higher α-glucosidase inhibition compared to that of acarbose used as standard (IC_50_ 358.75 ± 42.10 μg/mL) (Figure 3) and also to that of leaf extracts as reported in previous studies [9]. Fruit 3 showed the highest inhibition activity having the lowest value of IC_50_ (1.71 ± 0.23 μg/mL) (Figure 3). Several identified compounds (see following sections) have been previously demonstrated to be able to inhibit α-glucosidase, explaining the most of extracts activity. Recently it has been demonstrated that proanthocyanidins are potential natural α-glucosidase inhibitors; in fact, the *Chamaecyparis obtusa* var. *formosana* proanthocyanidins rich extracts demonstrated an IC_50_ comparable with our results (1 µg/mL) [26]. Moreover, tiliroside and flavonoid derivatives have been also demonstrated to be active as α-glucosidase inhibitors [27]. Further studied could be directed in the isolation and identification of minor compounds, not identified in this study, that might be also able to contribute to extract activity.

### 2.2. Chemical Profiles of Baobab Fruit Pulp

**Figure 4:**
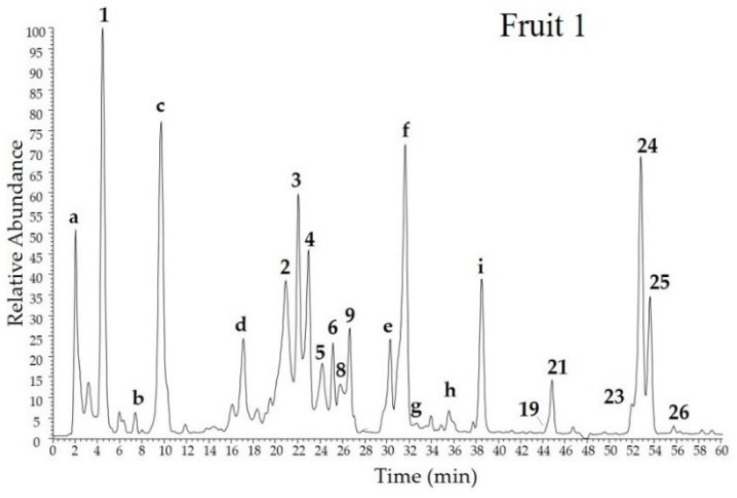

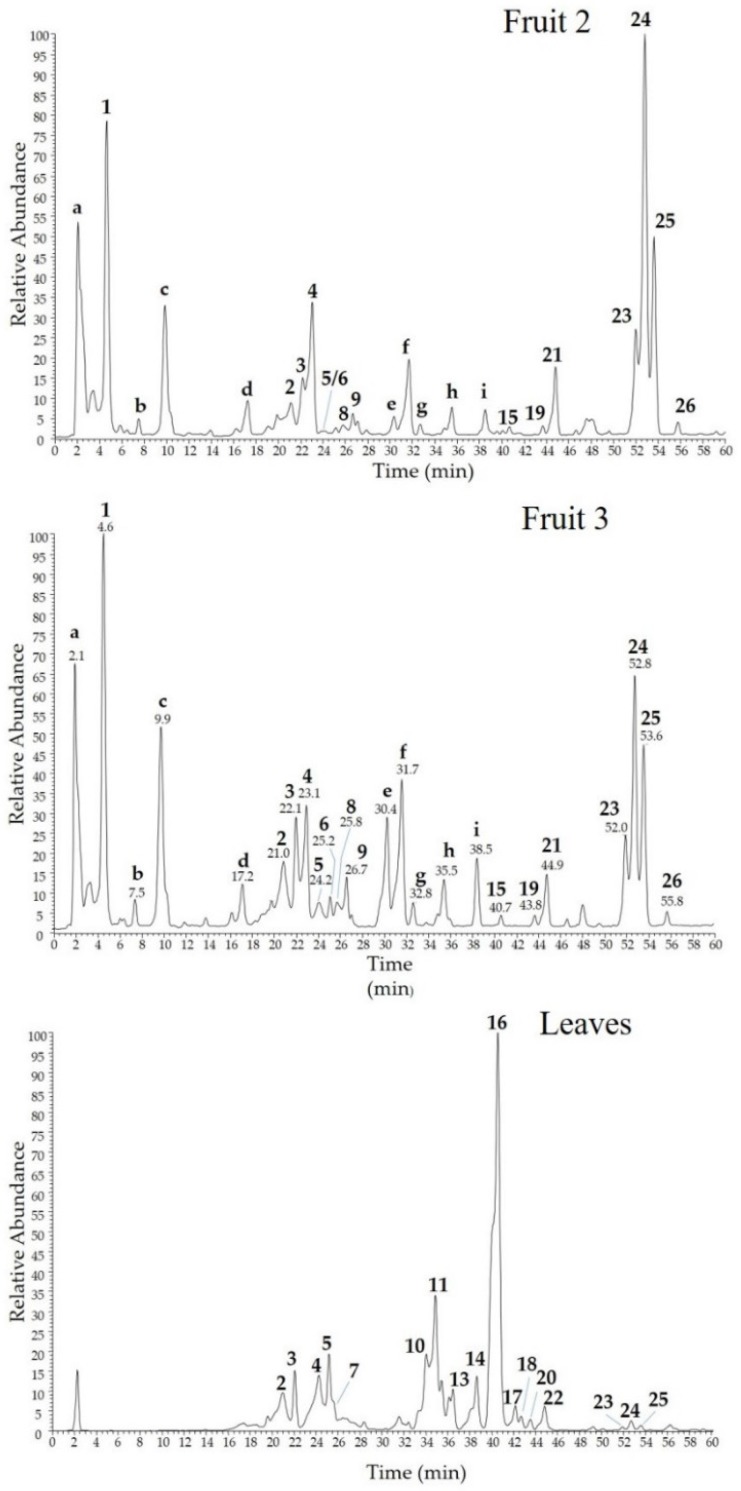
HPLC-ESI-MS/MS profiles of three Malian commercial samples of baobab pulp fruit (Fruits 1, 2, and 3) and leaves. Peak characteristics are showed in Table 2.

The analyses of bioactive compounds of baobab fruit pulp were performed by means of HPLC-PDA/UV-ESI-MS/MS experiments. The ESI-MS/MS chemical profiles of the three commercial investigated sample fruits are depicted in Figure 4. Phenol components were detected in two distinguished regions characterized by retention times (*t*_R_) 20–27 min for procyanidins and 44–57 min for flavonols.

As shown in Figure 4, all the fruit pulp extracts had a very similar composition. In total, fifteen compounds were identified by comparison of chromatographic and spectral data (Table 2) with those reported in the literature. Compounds **8**, **15**, **19**, **21**, and **24**–**26** were also compared with authentic reference standards available in our laboratory chemical library and previously isolated and characterized by 1D and 2D NMR experiments from plant materials. All the identified compounds, except for **1** that was recognised as citric acid, belong to the class of phenolics, in particular: one phenolic acid, feruloylquinic acid (**4**); two flavan-3-ols, catechin (**8**) and epicatechin (**9**) and their oligomers procyanidin dimer I (**2**) and II (**3**), procyanidin trimer I (**5**) and II (**6**); six flavonol glycosides, quercetin 3-*O*-glucoside (**15**), kaempferol 3-*O*-galactoside (**19**), kaempferol 3-*O*-glucoside (**21**), tiliroside I (**24**) and II (**25**) and their isomer (**23**); one flavonol aglycone, kaempferol (**26**).

Even though our attention was focused on secondary metabolites, among most polar compounds we underlined the presence of citric acid (**1**, *t*_R_ = 4.6 min), as a very known and abundant component of baobab fruit. Fragmentation pattern of deprotonated molecule [M − H]^−^ at *m*/*z* 191, showing diagnostic product ions at *m*/*z* 173, 155, 129, 111, and 87 generated by the losses of carboxylic moiety and water molecules, are in agreement with those reported in the literature [28]. According to previous studies on baobab fruit pulp from Cameroon [14] and from Malawi [6], Malian baobab fruit pulp contains proanthocyanidins in dymeric (**2** and **3**, *t*_R_ = 21.0 and 22.1 min) and trimeric forms (**5** and **6**, *t*_R_ = 24.2 and 25.2 min, respectively), together with relative monomers catechin (**8**, *t*_R_ = 25.8 min) and epicatechin (**9**, *t*_R_ = 26.7 min), all characterized by UV spectra with two strong absorption peaks at 243–247 and 279 nm. Compounds **8** and **9** showed the same molecular deprotonated ion [M − H]^−^ at *m*/*z* 289 and a formate adduct at *m*/*z* 335. The fragmentation of [M − H]^−^ parent ions in both cases generated typical fragments at *m*/*z* 245 and 205, consistent with previously reported data [29]. Furthermore, compound **8** was confirmed by injection of an authentic standard. The presence of two B-type procyanidin dimers (in which monomeric flavan-3-ol units are linked by a C4–C8 or C4–C6 bond) was indicated by the presence in the full mass spectra of deprotonated molecules at *m*/*z* 577 (peaks **2** and **3**) and in the MS/MS of base peak ions at *m*/*z* 425 together with other typical product ions at *m*/*z* 451, 407, 289, the last one corresponding to monomer catechin/epicatechin. Based on these data, we were not able to distinguish between various isomers, such as reported by Sokeng et al. [14]. In a previous study [6] procyanidin B2 was reported as constituent of baobab fruit from Malawi. Similarly, UV data and MS/MS experiments were useful to detect the presence of procyanidin trimers I and II (peaks **5** and **6**), showing [M − H]^−^ ions at *m*/*z* 865 and diagnostic product ions at *m*/*z* 847, 739, 713, 695 (base peak ion), 577, 451, 407, and 287, but it was not possible to determine the exact structures between different existing isomers, according to evidences reported by Sokeng et al. [14]. The full mass spectrum of compound **4** (*t*_R_ = 23.1 min) showed a formate adduct [M + HCOO]^−^ at *m*/*z* 413, indicating the molecular weight 368 amu. MS/MS experiment evidenced the presence of a product ion at *m*/*z* 191 that, according to results reported by Sokeng et al. [14], can be ascribable to a quinic acid residue generated by the loss of a feruloyl acid unit, suggesting compound **4** to be a feruloylquinic acid. Compounds **15**, **19**, **21**, and **23**–**26** are flavonol monoglycosides, as evidenced by UV data (Table 2). With the exception of compound **15** (*t*_R_ = 40.7 min), in which the aglycone is represented by quercetin, as evidenced by product ion at *m*/*z* 301 in the MS/MS, all the flavonoids are glycosides of kaempferol (base peak ions at *m*/*z* at 285). Sugar moieties are represented in all cases by a hexose unit, as deduced by MS/MS ions due to the loss of 162 amu, identified as glucose, except for **19** in which a galactose was linked to the aglycone. Moreover, compounds **23**–**25** (*t*_R_ = 52.0, 52.8, and 53.6 min, respectively) showed an initial loss of a coumaroyl residue ([M − H − 146]^−^ at *m*/*z* 447) linked to the glucose. All three molecules showed the same molecular weight ([M − H]^−^ at *m*/*z* 593), indicating the occurrence of three isomers. The injections of an authentic standard afforded to identified compounds **24** and **25** as *cis* and *trans* isomers of kaempferol 3-*O*-(6-*p*-coumaroyl)-glucoside, also named tiliroside, while for the isomer **23** a different linkage position of coumaroyl moiety on sugar unit, not determinable by MS data, could be supposed. Thus, discussed compounds were identified as quercetin 3-*O*-glucoside (**15**), kaempferol 3-*O*-galactoside (**19**), kaempferol 3-*O*-glucoside (**21**), tiliroside I (**24**) and II (**25**) and their isomer (**23**). Compound **15** was not detected in Fruit 1. Finally, compound **26** (*t*_R_ = 55.8 min), showing a deprotonated molecule [M − H]^−^ at *m*/*z* 285 was identified as kaempferol. These findings were confirmed by injection of reference standards. Some compounds remained unidentified. All of them were not detectable in positive ion mode, but only in the negative one. Peak **a** was a complex mixture of very polar compounds, thus difficult to characterize. Peak **b** ([M − H]^−^ at *m*/*z* 173) showed a mass fragmentation pattern close to citric acid (**1**), leading to suppose the presence of an organic acid, but based only on UV and mass spectra it is not possible to deduce its structure. Peaks **c** ([M − H]^−^ at *m*/*z* 205), **d** ([M − H]^−^ at *m*/*z* 219), and **i** ([M − H]^−^ at *m*/*z* 247) did not show any UV absorption, while MS/MS experiments revealed also in this case product ions in common with citric acid, suggesting the presence of other organic acids. Peaks **e**–**h** occurred as formate adducts, that showed fragmentation pathways not useful for their identification.

In summary, the chemical analyses of bioactive molecules of Malian baobab fruit pulp revealed the presence of phenolic compounds, according to results reported by Sokeng et al. [14]. Compared to the mentioned study, performed on fruit pulp sample from Cameroon, the Malian fruit pulp analyzed in the present study, lacked of dicaffeoylquinic acid and apigenin glycosides, but contained in addition kaempferol 3-*O*-galactoside (**19**), quercetin 3-*O*-glucoside (**15**), and kaempferol (**26**). On the contrary, the chemical profile of a Nigerian baobab fruit pulp [13] resulted to be very different, with compounds belonging to iridoid, phenylethanoid, and hydrocinnamic acid glycosides, not detected in the Malian fruit samples.

Results of quantitative analysis are listed in Table 3. The Fruit 2 resulted the richest in phenol components, followed by Fruit 3 and Fruit 1. These findings can appear in contrast with results of antioxidant activity assays, that showed Fruit 3 having the highest antioxidant potential and RACI value. Even though Fruit 2 contained tiliroside in highest amount, Fruit 3 compared to Fruit 2 showed a double content in procyanidins, catechin, and epicatechin. On the other hand, although these compounds are most represented in Fruit 1, in this sample the contents of tiliroside, kaempferol, and kaempferol 3-*O*-glucoside are lower than those of Fruit 3 and Fruit 2, while quercetin 3-*O*-glucoside is almost absent. However, it is known that the biological activity of a complex mixture can be due to a synergistic effect of its constituents, thus it is difficult to ascertain with precision the most active molecules.

### 2.3. Chemical Profile of Baobab Leaves

The HPLC-ESI-MS/MS profile of baobab leaves, presented in Figure 4, showed a very rich phenolic content, with eighteen detected compounds, consisting of procyanidin dimers (**2** and **3**), procyanidin trimers (**5** and **6**), procyanidin tetramer (**7**), *O*-glycosides of apigenin (**10**), quercetin (**11**, **14**, **16** and **17**), and kaempferol (**13**, **18**, **20**, **23**–**25**), one *C*-glycoside vitexin or isovitexin (**12**) and the aglycone quercetin (**22**). All compounds were identified by comparison of elution order, UV spectra, full mass spectra and mass fragmentation patterns (Table 2) with data reported in the literature [14]. Procyanidins (**2**, **3**, **5**, and **6**, *t*_R_ = 21.0, 22.1, 24.3, and 25.2 min, respectively), tiliroside I and II (**24** and **25**, *t*_R_ = 52.7 and 53.6 min, respectively), and tiliroside isomer (**23**, *t*_R_ = 52.0 min) were also detected in the fruit samples, thus discussion of their identification was reported in the previous section. In addition to these compounds, other phenolic constituents were revealed in the leaves. The most representative component was compound **14** (*t*_R_ = 40.5 min), identified as rutin (λ_max_ 256 and 356 nm). The fragmentation pattern of parent ion ([M − H]^−^ at *m*/*z* 609) showed the loss of rhamnose ([M − H − 146]^−^) and successively of glucose ([M − H − 146 − 162]^−^) at *m*/*z* 463 and 301, respectively. The aglycone quercetin was shown by product ion at *m*/*z* 301. The structure was confirmed by injection of a reference standard. Other quercetin derivatives, showing ESI-MS/MS base peak at 301 *m*/*z*, were compounds **11**, **16**, and **17**. Compound **16** (*t*_R_ = 42.1 min) was glycosilated with a pentoside, as deduced by product ion [M − H − 132]^−^ at *m*/*z* 301 generated by fragmentation of parent ion [M − H]^−^ at *m*/*z* 434. Compound **11** (*t*_R_ = 35.0 min, [M − H]^−^ at *m*/*z* 755), was a triglycosilated quercetin, with a saccharide chain consisting of two deoxyhexose and one hexose, as deduced by product ions at *m*/*z* 609 ([M − H − 146]^−^), 463 ([M − H − 146 − 146]^−^), and 301 ([M − H − 146 − 146 − 162]^−^). Based on MS/MS data it is no possible to assign the exact position and the identity of sugar units, but in a previous study Sokeng et al. [14] assigned the same compound to quercetin 3-*O*-(2,6-di-*O*-rhamnosyl)-glucoside. The full mass spectrum of compound **17** (*t*_R_ = 42.7 min) showed a deprotonated molecule [M − H]^−^ at *m*/*z* 607. MS/MS experiments evidenced the presence of a peak at *m*/*z* 463 ([M − H − 144]^−^), ascribable to a 3-hydroxy-3-methylglutaryl moiety, confirmed by diagnostic fragments at ([M − H − 62]^−^) and 505 ([M − H − 102]^−^) [28]. The product ion at *m*/*z* 301 ([M − H − 144 − 162]^−^) suggested an *O*-glycosidic linkage between quercetin and a hexose. Thus, compound **17** was tentatively identified as quercetin 3-hydroxy-3-methylglutaryl-*O*-hexoside, in agreement with Sokeng et al. [14]. In addition, the quercetin aglycone was identified (**22**, *t*_R_ = 50.1 min) by comparison of a reference standard showing the same chromatographic and UV data, together with diagnostic fragments in the ESI-MS/MS experiments (product ions at *m*/*z* 273, 257, 151, and 121) [29,30].

Compounds **18** (*t*_R_ = 43.5 min) and **20** (*t*_R_ = 44.8 min) were two kaempferol derivative isomers that showed the same ion molecular peak ([M − H]^−^ at *m*/*z* 593) of tiliroside. The loss of 146 uma ([M − H − 146]^−^ at *m*/*z* 447) could be due to a deoxyhexose moiety or a *p*-coumaroyl unit, as observed for tiliroside. By the injection of reference standards, we excluded the presence of kaempferol 3-*O*-rutinoside and kaempferol 3-*O*-neohesperidoside. The product ion at *m*/*z* 285 (corresponding to deprotonated kaempferol) was generated by the loss of one hexose, thus peaks **18** and **20** could be assigned to two isomers of tiliroside, but it is no possible to assign the right structures. Compound **13** (*t*_R_ = 38.6 min) showed a deprotonated molecule [M − H]^−^ at *m*/*z* 739, suggesting one more deoxyhexose or one *p*-coumaroyl residue compared to **18** and **20**. Indeed, the fragmentation pattern of parent ion was characterized by product ions at *m*/*z* 593 ([M − H − 146]^−^ and 285 ([M − H − 146 − 146 − 162]^−^). In a recent study, the same compound was detected in the baobab leaves and it was identified as kaempferol 3-*O*-(4′′-rhamnosyl)-neohesperidoside [14], even though it was not isolated and identified by NMR techniques.

An oligomeric procyanidin (**7**) was identified by UV absorption (λ_max_ 238 and 279 nm) and ESI mass spectrum showing a deprotonated molecule [M − H]^−^ at *m*/*z* 1153 and product ions at *m*/*z* 865 and 577, typical of a tetramer formed by catechin/isocatechin monomers.

Taking together the results from LC-PDA/UV-MS/MS analysis, Malian baobab leaves showed a very similar profile compared to leaves from Cameroon [14], whereas the study of Nigerian baobab leaves reported the presence of phenolic compounds, but the profile was quite different with less detected compounds [9].

### 2.4. Volatile Composition of Baobab Fruit Pulp and Leaves

To the best of our knowledge there is no previous study on the spontaneous emission profile of volatile organic compounds (VOCs) from leaves and fruit pulp of baobab. In total 59 compounds were identified with percentages of identification ranged from 85.5 to 97.8% and they are reported in Table 4. The identification of the constituents was based on the comparison of their retention time with those of pure authentic samples, comparing their linear indices (LRI) relative to a series of *n*-hydrocarbons, and on computer matching against commercial (NIST 98 [31] and ADAMS [32]) and also made possible by the use of a homemade library of mass spectra built up from pure substances and components of known oils, and MS literature data.

Leaf emission profile is mainly rich in sesquiterpene hydrocarbons (38.6%) with β-caryophyllene accounting for 24% but the content of apocarotenoids compounds (18.4%) is also remarkable.

Fruit 2 and 3 pulp emissions are quite similar: both are rich in anethole (31.6 and 24.0% respectively), a phenylpropanoid representing the most abundant identified compound; sesquiterpene hydrocarbons (3.8 and 24.8% respectively), oxygenated monoterpenes (15.4 and 20.8% respectively), and non terpene derivatives (31.6 and 15.1% respectively) are also abundant in pulp Fruit 2 and 3. Fruit 1 pulp emission is mainly rich in sesquiterpene hydrocarbons (56.5%) followed by non terpene derivatives (16.6%) and oxygenated monoterpenes (12.1%).

Low molecular weight non terpene derivatives were detected only in the leaves, while in all samples, among the main sesquiterpene hydrocarbons β-caryophyllene, longifolene, *α*-copaene and *α*-cedrene were the most abundant. Among the oxygenated monoterpenes detected in fruit samples the most abundant were menthone, borneol, and menthol. The most representative oxygenated sesquiterpenes among the fruit pulps was cedrol.

## 3. Materials and Methods

### 3.1. Chemicals

Methanol and formic acid HPLC grade were purchased from VWR (Milano, Italy). HPLC grade water (18 mΩ) was prepared by a Mill-Ω purification system (Millipore Co., Bedford, MA, USA). *n*-Hexane and *n*-butanol were achieved from VWR. Chloroform, glacial acetic acid and methanol were purchased from Carlo Erba (Milano, Italy). The reagents as sodium acetate trihydrate, 2,4,6-tripyridyl-s-triazine (TPTZ), iron (III) chloride (FeCl_3_∙6H_2_O), Folin-Ciocalteu reagent, carbonate sodium, 1,1-diphenyl-2-picryl hydrazyl (DPPH) radical, β-carotene, linoleic acid, Tween 20, 6-hydroxy-2,5,7,8-tetramethylchroman-2-carboxylic acid (Trolox), gallic acid, butylated hydroxytoluene (BHT), β-nicotinamide adenine dinucleotide reduced form (NADH), phenazine methosulfate (PMS), nitrotetrazolium blue chloride (NBT), l-ascorbic acid, potassium persulfate, 2,2′-azino-bis(3-ethylbenzothiazoline-6-sulphonic acid) (ABTS), *α*-glucosidase, acarbose, potassium phosphate buffer solution, 4-nitrophenyl *α*-d-glucopyranoside were obtained from Sigma-Aldrich S.p.a. (Milano, Italy). Phenols reference standards were obtained in our laboratories by previous isolation from plant materials and characterization by 1D and 2D NMR experiments.

### 3.2. Plant Material and Sample Preparation

The three samples of *Adansonia digitata* dried and powdered fruit pulp were purchased in Mali from three different local markets: Missabagu, Baraoueli region (Fruit 1); Place Coura, Bandiagara region (Fruit 2); Niamakoro, Segou region (Fruit 3). Fruit 1 and Fruit 3 were uniform fine powders, whereas Fruit 2 was characterized by granular material with different particle size and including residues of peels. Leaves of *A. digitata* were purchased by Korotoumou Traoré (AIDEMET) at the Market of Badalabougou (Bamako, Mali) from the Women Herbalist Association and were collected from District of Ségou on Sectember 2018 (Bamako, Mali). The plant material was identified by Seydou Dembélé and a voucher specimen (number 2358) was deposited in the Herbarium of Département Médecine Traditionelle (Bamako, Mali).

The three samples (Fruits 1, 2, and 3, 50.0 g each) and dried and powdered baobab leaves (50.0 g) were subjected to extraction at room temperature under stirring, first with *n*-hexane and successively with methanol (1 g/5 mL of solvent) for three times, every 24 h. The methanol leaf extract was subjected to RP-18 Solid Phase Extraction, eluting with methanol-water solution (9:1) to remove chlorophylls. The obtained dried residue (6.0 g), together with fruit pulp methanol extracts (8.8, 9.2, and 9.1 g for Fruits 1, 2, and 3, respectively) were partitioned between *n*-butanol and water. The *n*-butanol extracts (918.8, 914.7, and 717.8 mg for Fruits 1, 2, and 3, respectively, and 2.0 g for leaves) were dissolved in methanol (2.5 mg/mL), centrifuged and the supernatants were subjected to analyses by HPLC-PDA/UV-ESI-MS/MS.

### 3.3. DPPH Free Radical Scavenging

The radical scavenging activity of baobab fruit pulps was evaluated with DPPH test. As described by Todaro et al. [33], 50 μL of different dilutions of Trolox or extract was added to 200 μL of methanol solution of DPPH (100 μM) in a 96 well plate. The absorbance was measured at 515 nm after 30 min of incubation in the dark at room temperature. Decreasing the absorbance of the DPPH solution indicates an increase in DPPH radical scavenging activity. Results were expressed as mg of Trolox Equivalent (mgTE)/g of dried extract. Each reaction was performed in triplicate.

### 3.4. ABTS Assay

The radical scavenging activity of baobab fruit pulps was also analyzed with the 2,2′-azinobis-(3-ethylbenzothiazoline-6-sulfonic acid) diammonium salt (ABTS) radical assay, as described by Russo et al. [34] The reaction between ABTS salt and potassium persulfate generates the ABTS^+^ radical after 12 h of incubation at room temperature. The reaction for scavenging cationic radical was monitored at 734 nm and the results were expressed as mg of Trolox Equivalent per gram of dried extract (mgTE/g).

### 3.5. Ferric Reducing Antioxidant Power Assay (FRAP)

The FRAP assay was performed to evaluate the reducing power of the samples. Briefly, 25 μL of extract/trolox was added to 225 μL of FRAP reagent. FRAP reagent was composed by acetate buffer 300 mM pH 3.6, FeCl_3_∙6H_2_O 20 mM in distilled water and TPTZ 10 mM in HCl 40 mM in a proportion of 10:1:1. The mixture was incubated at 37 °C for 40 min in the dark. The absorbance of the solution was measured at 593 nm. Each reaction was performed in triplicate. Trolox was used as standard and the results were expressed as mg of Trolox equivalent per gram of dried extract (mgTE/g) [35].

### 3.6. β-Carotene Bleaching Test (BCB)

The β-carotene bleaching method (BCB) was used to evaluate the capacity of the extracts to inhibit the lipid peroxidation [35]. The BHT was used as positive control. A stock solution of β-carotene/linoleic acid was made by dissolving 0.2 mg of β-carotene in 0.2 mL of chloroform, linoleic acid (20 mg), and Tween 20 (200 mg). The chloroform was completely removed by rotary evaporator and distilled water (50 mL) was added with oxygen. The resulting emulsion was vigorously stirred. Aliquots (950 μL) of the mixture were transferred to the test tubes containing 50 μL of sample (the final concentration for all tested samples was 0.1 mg/mL) or solvent as blank. BHT was used as positive control. 250 μL of this solution were transferred to a 96 well plate and it was placed at 50 °C for 3 h. The absorbance was monitored at 470 nm for 180 min and measured every 30 min; each sample was carried out in triplicate. Results were expressed as percentage of antioxidant activity (AA) measured on the basis of β-carotene bleaching inhibition and calculated as follows:

% AA = [1 − (A sample T_0′_ − A sample T_180′_)/(A blank T_0′_ − A blank T_180_)] × 100 (1)

### 3.7. Superoxide Free Radical Scavenging

The capacity to scavenge superoxide radical was evaluated spectrophotometrically according to a previously described procedure [34]. Superoxide radicals were generated by the phenazine methosulfate-β-nicotinamide adenine dinucleotide (PMS-NADH) system, as previously reported. Several dilutions of sample (50 μL) or phosphate buffer as control, NADH (50 μL) and NBT (150 μL) were added into the 96-well plate. The reaction was started by adding PMS (50 μL) to the mixture. The assay was conducted at room temperature at 560 nm for 2 min. For each extract, five different concentrations were tested. Results were expressed as IC_50_ and ascorbic acid was used as positive control.

### 3.8. α-Glucosidase Assay

The α-glucosidase inhibition assay was performed according to the procedure of Bisio et al. [36]. The tested extracts and acarbose, a widely used clinical antidiabetic drug and used as positive control, were dissolved in methanol-DMSO 10%. Different concentration of the extracts (40 μL) were added to 130 μL of potassium phosphate buffer solution (PPBS, 10 mM, pH 7) and 60 μL of substrate (4-nitrophenyl α-d-glucopyranoside 2.5 mM) in PPBS. The absorbance was measured at 405 nm (T_0_), then the reaction was initiated by the addition of 20 µL of α-glucosidase enzyme stock solution (0.28 units/mL in PPBS). The plates were incubated at 37 °C for 10 min. After incubation, the absorbance were determined again (T_10_). For the negative control wells, sample was substituted with solvent used to dissolve fruit extracts. The inhibition percentage was calculated in according to the following equation:

(2) $$\%\;inhibition=\left(1-\frac{A_{405}\;sample_{\;\left(T_{10\prime }-T_{0\prime }\right)}}{A_{405}\;negative\;control_{\;\left(T_{10\prime }-T_{0\prime }\right)}}\right)\times 100$$

The IC_50_ values were determined by GraphPad Prism 5 Software (San Diego, CA, USA). They were estimated by nonlinear curve-fitting and presented as their respective 95% confidence limits.

### 3.9. Total Phenolic Content (TPC)

The evaluation of the total polyphenolic content (TPC) was carried out by using Folin-Ciocalteu reagent [19]. The assay consisted in adding 425 μL of distilled water and 75 μL of gallic acid/extract to 500 μL of Folin-Ciocalteu reagent and 500 μL of Na_2_CO_3_ (10% *v*/*v* in H_2_O). The samples were vortexed and incubated for 1 h in the dark. After incubation the absorbance was measured at 723 nm. All the reactions were performed in triplicate. Gallic acid was used as standard to build the standard curve and the results were expressed as mg gallic acid equivalent (GAE)/g of dried extract [19].

### 3.10. Chemical Characterization and Quantification of Phenols by HPLC-PDA/UV-ESI-MS/MS

HPLC-PDA/UV-ESI-MS/MS analyses were performed using a Surveyor LC pump, a Surveyor autosampler, coupled with a Surveyor PDA/UV detector, and a LCQ Advantage ion trap mass spectrometer (ThermoFinnigan, San Jose, CA, USA) equipped with Xcalibur 3.1 software. Chromatographic analyses were achieved using a 4.6 × 150 mm, 5 µm, Luna (C-18) column (Phenomenex, Bologna, Italy) by triplicate injections. Elution was executed using a mixture of methanol (solvent A) and formic acid in water 0.1% *v*/*v* (solvent B) as an eluent and a linear gradient of increasing 5% to 65% A was developed within 60 min. The column was successively washed for 15 min with methanol and equilibrated with 5% A for 10 min. The flow rate was 0.8 mL/min, with a splitting system of 2:8 to MS detector (160 μL/min) and PDA detector (640 μL/min), respectively. The volume of the injected extract solutions was 20 μL. All analyses were performed with an ESI interface in the negative mode in a scan range of *m*/*z* 150–2000 and the ionization conditions were optimized as follows: capillary temperature, 270 °C; source voltage, 4.50 kV; capillary voltage, −16.0 V; tube lens offset, −5.0 V; sheath gas flow rate, 60.00 arbitrary units; auxiliary gas flow rate, 3.00 arbitrary units; spray voltage, 4.50 kV [37]. N_2_ was used as the sheath and auxiliary gas. In the ESI-MS/MS experiments normalized collision energy 35.0% was used. PDA/UV data were recorded with 200–600 nm as detection wavelength range and 254, 280, and 325 nm preferential channels.

The amounts of main phenol components detected in baobab fruit pulps were determined constructing a calibration curve using the following compounds as external standard: procyanidin B3 and catechin (concentration range 1.00–100.0 µg/mL), tiliroside (concentration range 10.00–500.00 µg/mL), kaempferol, and kaempferol glucoside (concentration range 0.05–5.00 µg/mL), chlorogenic cid (concentration range 1.00–100.00 µg/mL). At least four different concentrations of standard methanol solutions were prepared by serial dilution from stock solution (1 mg/mL) and analysed by triplicate injections. The calibration curve was generated by using concentration (mg/mL) with respect to the area obtained from the integration of the MS base peak [M − H]^−^ of each standard. The relation between variables was analyzed using linear simple correlation. For the linear regression of the standards, *R*^2^ was 0.9994 for procyanidin B3, 0.9943 for tiliroside, 0.9965 for kaempferol, 0.9961 for kaempferol glucoside, 0.9995 for catechin, and 0.9956 for chlorogenic acid. Accuracy was evaluated by spiking the samples with known amount of standard solutions. Precision was verified by analyzing samples 3 times on different days, and the relative standard deviation was always below 20%. The phenol amounts were obtained by using a GraphPad Software Prism 6.0 and finally expressed as μg/g of dried pulp fruit.

### 3.11. Analysis of VOCs by HS-SPME-GC/MS

The analysis of volatile compounds was performed by the Solid-Phase Micro-Extraction (SPME) technique, using a Supelco SPME devices (Sigma-Aldrich) coated with polydimethylsiloxane (PDMS, 100 μm). A portion of the dried pulp of each fruit and of dried leaves was put into a 50 mL flask and allowed to equilibrate for 30 min at room temperature. The fiber, previously conditioned according to the manufacturer recommendations, was exposed to the headspace of the sample respectively for 20 (pulp) and 30 (leaves) min. Sampling was performed in an air-conditioned room (22 ± 1 °C) to guarantee a stable temperature during sampling. After the sampling time, the fiber was withdrawn into the needle, then transferred immediately to the injection port of the GC/MS, where the fiber was desorbed with a splitless injection method. Gas chromatography-electron impact mass spectrometry (GC/EI-MS) analyses were performed with a CP-3800 gas chromatograph (Varian, Palo Alto, CA, USA) equipped with a DB-5 capillary column (30 m × 0.25 mm; coating thickness 0.25 μm) and a Varian Saturn 2000 ion trap mass detector. Analytical conditions: injector and transfer line temperatures 250 °C and 240 °C, respectively; oven temperature programmed from 60 to 240 °C at 3 °C/min; carrier gas helium at 1 mL/min.

### 3.12. Statistical Analysis

Date are expressed as mean ± standard deviation (Mean ± SD). Statistical analysis was performed by analysis of variance (one-way ANOVA) followed by Tukey test using GraphPad Prism 5 Software, Inc. (San Diego, CA, USA) and *p* value of 0.05 or less was considered as statistically significant. All measurements were performed by using SPECTROstar^Nano^ (BMG Labtech, Ortenberg, Germany).

## 4. Conclusions

Our results suggested that Malian baobab fruit pulp and leaves are rich source of procyanidins and flavonol glycosides, with a different in qualitative composition compared to those of other African countries, indicating that growing region affected their chemical profile. Thus additional studies on samples from different countries could enrich the knowledge of this fruit with a great potential as functional food.

Antioxidant and α-glucosidase inhibition activity assays confirmed the classification of baobab as “super fruit”, probably the most important foodstuff of the Malian flora. The presence of potential nutraceuticals is suitable and promising for the development of safe food products and plant additives, in a country as Mali where the fight against malnutrition is still an essential challenge.

## Figures and Tables

**Table 1:** Antioxidant activity of three different *n*-butanol extracts of baobab fruit pulp.

	TPC (mgGAE/g) *	DPPH (mgTE/g) **	ABTS (mgTE/g) **	FRAP (mgTE/g) **	BCB (%AA) ***	SO (IC_50_) ****
Fruit 1	148.19 ± 6.40 ^a^	355.42 ± 2.76 ^a^	667.38 ± 34.56 ^a^	439.94 ± 26.02 ^a^	12.01 ± 3.21 ^a^	0.18 ± 0.02 ^a^
Fruit 2	120.07 ± 4.67 ^b^	322.65 ± 7.95 ^a^	654.71 ± 26.72 ^a^	293.99 ± 14.58 ^b^	57.19 ± 2.04 ^b^	0.89 ± 0.09 ^a^
Fruit 3	161.40 ± 2.82 ^c^	392.22 ± 28.13 ^b^	799.44 ± 35.96 ^b^	458.50 ± 23.41 ^a^	68.83 ± 0.38 ^b^	0.55 ± 0.002 ^a^

* TPC: total polyphenol content expressed as milligrams of gallic acid per grams of extract; ** DPPH: 2,2-diphenyl-1-picrylhydrazyl; ABTS: 2,2′-azinobis-(3-ethylbenzothiazoline-6-sulfonic acid) diammonium salt; FRAP: ferric reducing antioxidant power expressed as milligrams of Trolox equivalents per grams of extract; *** BCB: β-carotene bleaching assay expressed as antioxidant activity at 0.5 mg/mL; **** SO: superoxide anion expressed as concentration required for 50% inhibition. Experiments were carried out in triplicate and data were reported as mean ± SD. Significant differences (*p* < 0.05) are highlighted with different letters (a, b and c).

**Table 2:** Mass/UV spectral data and retention time (*t*_R_) of compounds **1**–**26** detected in the baobab fruit pulp (F) and leaves (L). Compound numbers correspond with peak numbers in Figure 4. Compounds are listed in order of increasing *t*_R_.

Peak	Compound	*t*_R_ (min)	M	[M + HCOO]^−^	[M − H]^−^	ESI-MS/MS Ions (*m*/*z*) *	UV (λ_max_)	Organ
	Organic acids							
**1**	citric acid	4.6	192		191	173, 129, **111**, 87	212	F
	Phenolic compounds							
**2**	procyanidin dimer I	21.0	578		577	451, **425**, 407, 289	246, 279	F, L
**3**	procyanidin dimer II	22.1	578		577	451, **425**, 407, 289	245, 279	F, L
**4**	feruloylquinic acid	23.1	368	413 **	367	**367**, 191	248, 279	F
**5**	procyanidin trimer I	24.2	866		865	847, 739, 713, **695**, 577, 451, 407, 287	247, 279	F, L
**6**	procyanidin trimer II	25.2	866		865	847, 739, 713, **695**, 577, 451, 407, 287	246, 279	F, L
**7**	procyanidin tetramer	25.5	1154		1153	1001, **865**, 577, 455	238, 279	L
**8**	catechin	25.8	290	335	289	**245**, 205, 179	245, 279	F
**9**	epicatechin	26.7	290	335	289	**245**, 205, 179	243, 279	F
**10**	apigenin *O*-pentoside	34.0	448	493	447	357, **327**, 285	242, 270, 349	L
**11**	quercetin glycoside ***	35.0	756		755	737, 609, 591, 489, **301**	244, 268, 352	L
**12**	vitexin/isovitexin	36.5	432		431	341, **311**	243, 273, 331	L
**13**	kaempferol glycoside ****	38.6	740		739	593, 575, **285**	244, 278	L
**14**	rutin	40.5	610		609	463, 343, **301**, 271, 255, 179	256, 356	L
**15**	quercetin 3-*O*-glucoside	40.7	464		463	445, **301**, 179	246, 278	F
**16**	quercetin pentoside	42.1	434		433	**301**, 255, 179	248, 268, 355	L
**17**	quercetin 3-hydroxy-3-methylglutaryl-*O*-hexoside	42.7	608		607	545, 505, **463**, 301, 179	247, 273, 344	L
**18**	kaempferol glycoside I	43.5	594	639	593	447, **285**	247, 270, 315	L
**19**	kaempferol 3-*O*-galactoside	43.8	448		447	327, **285**	247, 278	F
**20**	kaempferol glycoside II	44.8	594	639	593	447, **285**	248, 269, 315	L
**21**	kaempferol 3-*O*-glucoside	44.9	448		447	327, **285**	247, 278	F
**22**	quercetin	50.1	302		301	273, 257, 229, **179**, 151, 121, 107	277	L
**23**	tiliroside isomer	52.0	594		593	447, 429, **285**	270, 312	F, L
**24**	tiliroside I	52.8	594		593	447, 429, **285**	268, 287, 315	F, L
**25**	tiliroside II	53.6	594		593	447, 429, **285**	269, 312	F, L
**26**	kaempferol	55.8	286		285	**285**, 257, 229	277	F

* The base ion peaks generated in the ESI-MS/MS experiments are shown in bold; ** Product ions were generated by fragmentation of [M + HCOO]^−^; *** Identified as quercetin 3-*O*-(2,6-di-*O*-rhamnosyl)-glucoside by Sokeng et al. [14]; **** Identified as kaempferol 3-*O*-(4′′-rhamnosyl)-neohesperidoside by Sokeng et al. [14].

**Table 3:** Quantitative amount (μg/g ± standard deviation of dried fruit pulp) of phenol constituents detected in baobab fruit pulp. Compound numbers (in bold) correspond with peak numbers in Figure 4.

	Peak	Fruit 1	Fruit 2	Fruit 3
procyanidins	**2**, **3**, **5**, **6**	1.07 ± 0.01	0.228 ± 0.002	0.50 ± 0.09
tiliroside I, II and isomer	**23**, **24**, **25**	17.4 ± 0.6	33 ± 2	23 ± 2
kaempferol	**26**	0.010 ± 0.001	0.017 ± 0.001	0.020 ± 0.002
kaempferol 3-*O*-glucoside	**21**	0.13 ± 0.02	0.18 ± 0.01	0.144 ± 0.002
catechin and epicatechin	**8**, **9**	4.5 ± 0.1	1.28 ± 0.04	2.5 ± 0.2
quercetin 3-*O*-glucoside	**15**	nd	0.010 ± 0.001	0.010 ± 0.001
feruloylquinic acid	**4**	0.29 ± 0.01	0.27 ± 0.01	0.22 ± 0.01
Total		23 ± 1	35 ± 2	26 ± 2

**Table 4:** Total volatile organic compound (VOC) profile for the baobab samples by HS-SPME-GC/MS.

N.	*t*_R_	LRI	Component	Relative Content %			
				Leaves	Fruit 1	Fruit 2	Fruit 3
1	2.01	736	isopentyl alcohol	tr	-	-	-
2	2.56	800	hexanal	tr	-	-	-
3	3.01	834	isovaleric acid	tr	-	-	-
4	3.28	853	(*E*)-3-hexen-1-ol	2.6	-	-	-
5	4.72	926	tricyclene	tr	-	-	-
6	5.36	953	1-hexanol	1.7	-	-	-
7	5.44	961	benzaldehyde	0.5	-	-	-
8	5.79	980	β-pinene	0.3	-	-	-
9	5.85	985	1-octen-3-ol	0.1	-	-	-
10	6.05	987	6-methyl-5-hepten-2-one	4.8	tr	tr	tr
11	6.72	1011	δ-3-canene	0.1	-	-	-
12	7.10	1026	1-*p*-menthene	1.6	-	-	-
13	7.23	1031	3-ethyl-1-hexanol	tr	-	-	-
14	7.30	1032	limonene	1.2	6.1	-	-
15	7.37	1035	1,8-cineole	tr	1.0	2.0	tr
16	8.80	1079	*trans*-linalool oxide (furanoid)	1.9	1.2	0.3	tr
17	9.37	1090	*cis*-linalool oxide (furanoid)	1.0	1.0	0.2	-
18	9.83	1098	linalool	1.7	4.6	-	tr
19	9.97	1102	nonanal	0.4	6.5	6.4	4.8
20	10.11	1105	α-thujone	4.2	-	-	tr
21	10.53	1110	phenyl ethyl alcohol	0.8	-	-	-
22	11.51	1143	camphor	-	tr	0.5	1.9
23	11.80	1151	ethyl hexyl acetate	0.1	-	-	-
24	11.91	1154	menthone	-	4.3	5.0	7.4
25	12.31	1164	isomenthone	-	tr	2.0	2.5
26	12.43	1165	borneol	-	1.9	1.7	2.4
27	12.73	1173	menthol	-	tr	4.8	3.9
28	12.91	1177	*trans*-linalool oxide (pyranoid)	0.7	-	-	-
29	13.50	1190	α-terpineol	tr	-	-	-
30	13.81	1200	safranal	0.9	tr	0.4	-
31	14.09	1204	decanal	0.6	tr	3.3	5.3
32	14.69	1217	β-cyclocitral	2.5	-	-	-
33	15.73	1240	cuminaldheyde	-	-	1.7	1.3
34	16.25	1256	β-cyclo-homocitral	0.7	-	-	-
35	17.7	1283	(*E*)-anethole	-	tr	31.6	24.0
36	18.74	1312	2,3,4-trimethyl benzaldheyde	-	-	0.6	-
37	19.71	1340	δ-elemene	0.6	tr	tr	-
38	20.23	1351	δ-longipinene	0.1	-	-	-
39	21.34	1376	α-copaene	5.0	7.3	2.0	1.9
40	22.08	1391	β-elemene	0.7	-	-	-
41	22.45	1400	tetradecane	1.3	-	tr	-
42	22.54	1402	longifolene	0.7	17.9	5.7	4.6
43	22.87	1408	α-cedrene	-	4.4	1.1	3.5
44	23.18	1418	β-caryophyllene	24.0	21.2	4.3	9.3
45	23.57	1433	γ-elemene	4.6	-	-	1.9
46	23.96	1439	trans-α-bergamotene	-	-	-	0.7
47	24.62	1454	α-humulene	1.0	-	-	-
48	24.76	1456	(*E*)-geranyl acetone	8.1	-	-	1.8
49	25.96	1483	ar-curcumene	-	1.6	-	tr
50	26.07	1485	(*E*)-β-ionone	6.2	-	-	-
51	26.36	1495	bicyclogermacrene	0.6	-	-	-
52	26.67	1500	*n*-pentadecane	-	-	1.1	2.3
53	27.00	1509	β-bisabolene	1.3	tr	0.5	1.0
54	27.56	1524	δ-cadinene	-	3.9	1.3	1.8
55	27.84	1536	dihydroactinidiolide	4.3	-	-	-
56	29.93	1581	caryophyllene oxide	1.2	tr	0.8	tr
57	30.68	1599	cedrol	-	6.2	2.4	5.0
58	34.45	1700	*n*-heptadecane	-	-	tr	-
59	39.87	1845	hexahydrofarnesyl acetone	0.5	-	-	-
Total				89.3	97.8	85.5	91.5
**Class of Compounds**				**Leaves**	**Fruit 1**	**Fruit 2**	**Fruit 3**
monoterpene hydrocarbons				3.3	6.1	18.1	-
oxygenated monoterpenes				9.7	12.1	15.4	20.8
sesquiterpene hydrocarbons				38.6	56.5	3.8	24.8
oxygenated sesquiterpenes				6.0	6.3	16.2	5.1
non terpene derivatives				13.3	16.6	31.6	15.1
phenylpropanoids				-	0.1	0.4	24.0
apocarotenoids				18.4	0.1	18.1	1.8

LRI = linear retention index; tr = traces.
